# Untargeted metabolomics to understand the basis of phenotypic differences in amphotericin B-resistant *Leishmania* parasites

**DOI:** 10.12688/wellcomeopenres.15452.1

**Published:** 2019-11-13

**Authors:** Andrew W. Pountain, Michael P. Barrett

**Affiliations:** 1 Wellcome Center for Integrative Parasitology, University of Glasgow, Glasgow, G12 8TA, UK; 2 Department of Microbiology and Molecular Genetics, McGovern Medical School, University of Texas Health Science Center at Houston, Houston, Texas, 77030, USA; 3 Glasgow Polyomics, Wolfson Wohl Cancer Research Centre, University of Glasgow, Bearsden, Glasgow, G61 1QH, UK

**Keywords:** Leishmania, amphotericin B, drug resistance, metabolomics

## Abstract

**Background**: Protozoan *Leishmania* parasites are responsible for a range of clinical infections that represent a substantial challenge for global health. Amphotericin B (AmB) is increasingly used to treat *Leishmania* infection, so understanding the potential for resistance to this drug is an important priority. Previously we described four independently-derived AmB-resistant *L. mexicana* lines that exhibited resistance-associated genetic lesions resulting in altered sterol content. However, substantial phenotypic variation between these lines, including differences in virulence attributes, were not fully explained by these changes.

**Methods: **To identify alterations in cellular metabolism potentially related to phenotypic differences between wild-type and AmB-resistant lines, we extracted metabolites and performed untargeted metabolomics by liquid chromatography-mass spectrometry.

**Results: **We observed substantial differences in metabolite abundance between lines, arising in an apparently stochastic manner. Concerted remodeling of central carbon metabolism was not observed; however, in three lines, decreased abundance of several oligohexoses was observed. Given that the oligomannose mannogen is an important virulence factor in *Leishmania*, this could relate to loss of virulence in these lines. Increased abundance of the reduced forms of the oxidative stress-protective thiols trypanothione and glutathione was also observed in multiple lines.

**Conclusions: **This dataset will provide a useful resource for understanding the molecular basis of drug resistance in *Leishmania*, and suggests a role for metabolic changes separate from the primary mechanism of drug resistance in determining the phenotypic profile of parasite lines subjected to experimental selection of resistance.

## Introduction

Parasites of the genus *Leishmania* place a high burden on public health, with clinical manifestations ranging from self-healing cutaneous lesions to life-threatening visceral leishmaniasis. Efforts to control this disease rely heavily on chemotherapeutic agents, but the traditional front-line class, pentavalent antimonials, suffers from both high toxicity and increasing drug resistance ^1^. Another widely used compound, miltefosine, has also shown a trend of decreasing clinical efficacy ^2, 3^. In this context, the antifungal polyene amphotericin B (AmB) is increasingly the treatment of choice for leishmaniasis, with the introduction of liposomal formulations serving to increase efficacy and reduce adverse effects ^4^.

The mechanism of action of AmB is not fully understood but involves specific binding to ergosterol within parasite and fungal membranes, leading to disruption of membrane function either through the formation of aqueous pores or by sequestering ergosterol within the membrane ^5, 6^. Resistance to this compound is not yet widespread in *Leishmania* populations but has been documented both in clinical isolates ^7– 9^ and through experimental selection or genetic manipulation ^10– 17^. However, the introduction of single-dose regimens of liposomal AmB risks enhancing conditions conducive to resistance selection in the field. Resistance is generally associated with mutations in the ergosterol biosynthesis pathway. Recently, we described four AmB-resistant *L. mexicana* lines selected independently *in vitro* and subjected to phenotypic, genomic and transcriptomic characterization ^15^. All four demonstrated lesions within the ergosterol biosynthesis pathway. Specifically, AmBRA/cl1 demonstrated a point mutation in the gene sterol C5-desaturase ( *LmxM.23.1300*), whereas AmBRB/cl2, AmBRC/cl3 and AmBRD/cl2 all demonstrated structural variants leading to greatly reduced expression of sterol C24-methyltransferase genes ( *LmxM.36.2380* and *LmxM.36.2390*). Sterol analysis by gas chromatography-mass spectrometry revealed corresponding changes in the sterol composition of these strains, and genetic complementation studies demonstrated that both genes played key roles in AmB resistance. In addition, resistance in AmBRB/cl2 was partially the result of deletion of the miltefosine transporter gene ( *LmxM.13.1530*), also associated with miltefosine cross-resistance in this line. Loss of miltefosine transporter function was previously reported in an AmB-resistant line ^13^, and an RNAi knockdown screen in the related parasite *Trypanosoma brucei* revealed that reduced expression of the *T. brucei* ortholog of *LmxM.13.1530* is also associated with AmB resistance ^18^.

Whilst this study confirms an important role for sterol changes in AmB resistance, other studies suggest that additional cellular processes may play a role. Increased expression of genes associated with defense against reactive oxygen species (ROS) has been found in drug-resistant *L. infantum* ^19^, and overexpression of genes of the pentose phosphate pathway (PPP, an important source of NADPH reducing equivalents) decreased AmB sensitivity ^20^. Whilst previous studies in *Leishmania* ^14^ and *Candida albicans* ^21^ have associated AmB resistance with increased sensitivity to oxidative stress, no strain in our study ^15^ exhibited greatly increased sensitivity to glucose oxidase (a source of H _2_O _2_) nor menadione (which leads to the generation of intracellular ROS). Therefore, it is possible that the lack of substantial ROS hypersensitivity in these lines is explained by compensatory metabolic changes in redox metabolism.

Another unanswered question relates to virulence phenotypes in the AmB-resistant lines. Only one of the four lines, AmBRC/cl3, retained similar capacity to infect macrophages to that of the parental wild-type line (as well as retaining virulence in a mouse model of cutaneous infection). However, resistance in this line was associated with a disruption of sterol C24-methyltransferase function highly similar to that found in AmBRB/cl2 and AmBRD/cl2 ^15^. This suggests that loss of virulence may result from a separate change, possibly arising spontaneously during adaptation to long-term culture *in vitro*. Genomic and transcriptomic analyses alone did not provide a clear indication of any such mechanism.

Therefore, we wished to determine whether some of these phenotypes resulted from changes in cellular metabolism. We performed untargeted metabolomics analysis using liquid chromatography-mass spectrometry (LC-MS), to detect differences between AmB-resistant lines and the parental wild-type line. Whilst numerous changes were observed, many of these were not conserved between lines, suggesting a stochastic origin rather than selection in response to drug pressure. Nevertheless, evidence of adaptation to oxidative stress was observed, and changes to oligosaccharide abundance suggest a possible metabolic basis for the variation in virulence between these lines.

## Results

Metabolomics analysis reveals evidence of stochastic changes in metabolism in independently-derived AmB-resistant *L. mexicana* strains

**Figure 1.**
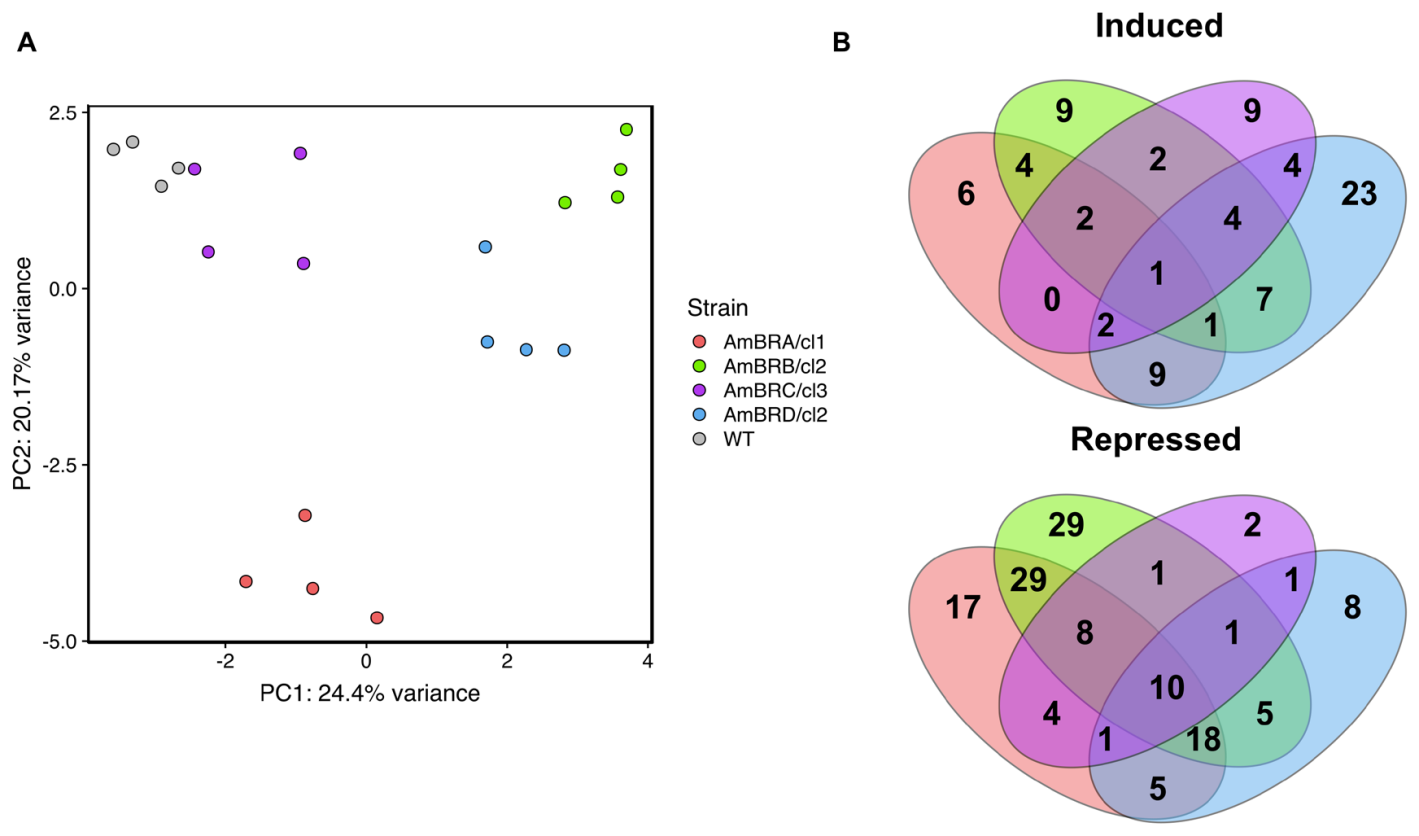
Overall trends in untargeted metabolomics data. **A**) Principal component analysis performed on range-scaled data. The 319 identified metabolites that met filtering criteria (see Methods) were all included. **B**) Venn diagrams showing overlap of metabolites induced or repressed across different AmB-resistant lines. Metabolites included are those with Benjamini-Hochberg-corrected p-value (q-value) < 0.05 for ANOVA, and a Tukey’s Honest Significant Difference test comparing to wild-type with p-value < 0.05.

After filtering data for peak quality, 319 metabolites were identified on the basis of mass, of which 232 showed an effect of strain on metabolite abundance (one-way ANOVA with Benjamini Hochberg correction, P < 0.05) ^22^. Within these metabolites, a Tukey’s Honest Significant Difference test was performed to determine metabolites significantly differing (P < 0.05) from the wild-type parental line. Limited overlap was evident between AmBR strains, and principal component analysis further demonstrated a lack of similarity of lines ( Figure 1A). Of 83 metabolites showing increased abundance in at least one AmBR strain relative to wild-type, 47 (57%) were unique to an individual strain, with 56 out of 139 metabolites (40%) showing repression in one strain only ( Figure 1B). Only one metabolite, succinate, showed universally increased abundance in AmBR strains relative to the parental line, albeit modestly (30–57% increase). These data suggest that the majority of metabolic phenotypes are governed by stochastic changes during cell culture, as opposed to the effects of selection pressure with AmB.

### Changes in carbohydrate and energy metabolism

**Figure 2.**
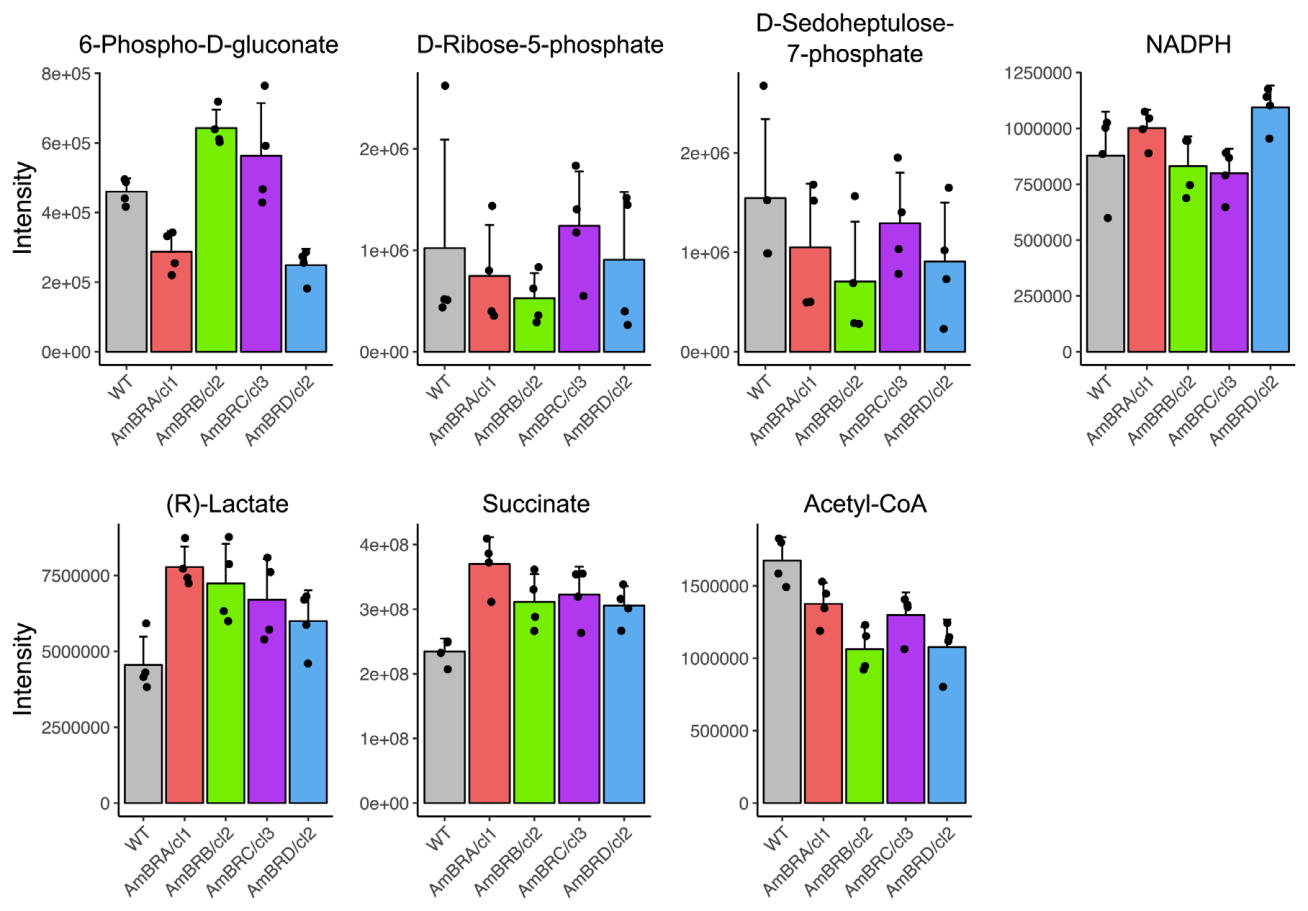
Metabolite intensity plots for metabolites involved in carbohydrate and energy metabolism. Mean intensities across four biological replicates are shown, with error bars representing standard deviation. Black points represent intensities of individual replicates.

Since overexpression of PPP enzymes leads to greater tolerance of oxidative stress and AmB resistance ^20^, we investigated whether AmBR lines had a higher abundance of PPP intermediates. Amongst detectable PPP metabolites, none showed a higher abundance compared to WT, and some decreases were noted ( Figure 2). This was reflected in the fact that NADPH abundance was unchanged ( Figure 2). As repression of mitochondrial activity in *Candida albicans* fungi leads to reduced ROS generation in response to AmB treatment ^23^, we also looked for evidence of broader restructuring of energy metabolism. Whilst there was little evidence of generally altered abundance of intermediates in either glycolysis or the tricarboxylic acid (TCA) cycle in any AmBR line, both lactate and succinate showed increases across all lines ( Figure 2). Succinate is an end product of fermentative metabolism in *Leishmania* ^24^ and given the general trend towards decreased abundance of acetyl CoA (the first committed step of the TCA cycle), this may suggest a modest shift towards fermentation over respiration in these lines.

**Figure 3.**
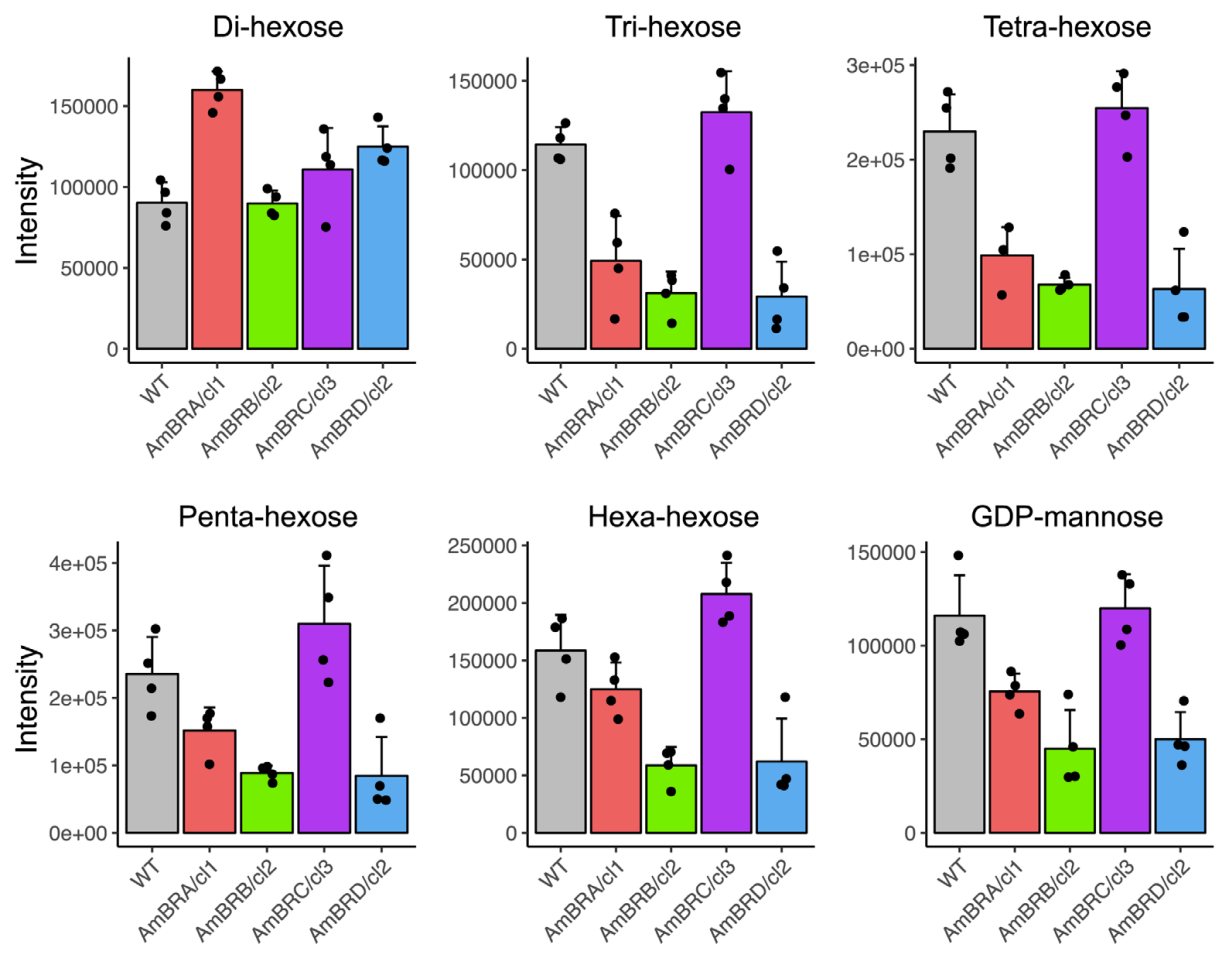
Metabolite intensity plots for metabolites relating to mannogen synthesis. Metabolites were identified as di- to hexa-hexoses on the basis of mass, and may be oligomannans, as described in the text. Mean intensities across four biological replicates are shown, with error bars representing standard deviation. Black points represent intensities of individual replicates.

Therefore, changes in central carbon metabolism were confined largely to individual metabolites rather than whole pathways. In contrast, amongst putative oligosaccharides identified (3–6 hexose units – longer polymers extend beyond the detectable size range using this platform), there was a consistent pattern of decreased abundance in three out of four lines (AmBRA/cl1, AmBRB/c2 and AmBRD/c2), whilst no changes were observed in AmBRC/cl3 ( Figure 3). As different hexose isomers are indistinguishable by mass and standards for these compounds were not included, we are unable to determine explicitly the identity of these oligohexoses, but since a mannose polymer, mannogen, is a highly abundant storage carbohydrate in *Leishmania* ^25^, it is likely that these are oligomannoses. Further evidence of this can be seen in the similar pattern of decreases in GDP-mannose, which is mannose donor during mannogen synthesis ^26^. While this pattern did not extend to a putative di-hexose, this could be a disaccharide other than di-mannose (although the retention time did not match to the isomeric disaccharide sucrose, included as a standard).

Several metabolites relating to lipid metabolism were detected, including 23 with differential expression compared to WT in at least one line ^22^. Given the abundance of isomers of lipid compounds, the lack of available standards, and the fact that many of these have very similar retention times, one cannot confidently assign identities to these compounds. Nevertheless, 18 showed altered abundance in AmBRB/cl2, of which 15 were decreased. As a homozygous deletion of the miltefosine transporter, a phospholipid flippase, was previously detected in this line ^15^, we note that several metabolites show greater changes in abundance in AmBRB/cl2 than in other lines.

### Changes in amino acid and polyamine metabolism

**Figure 4.**
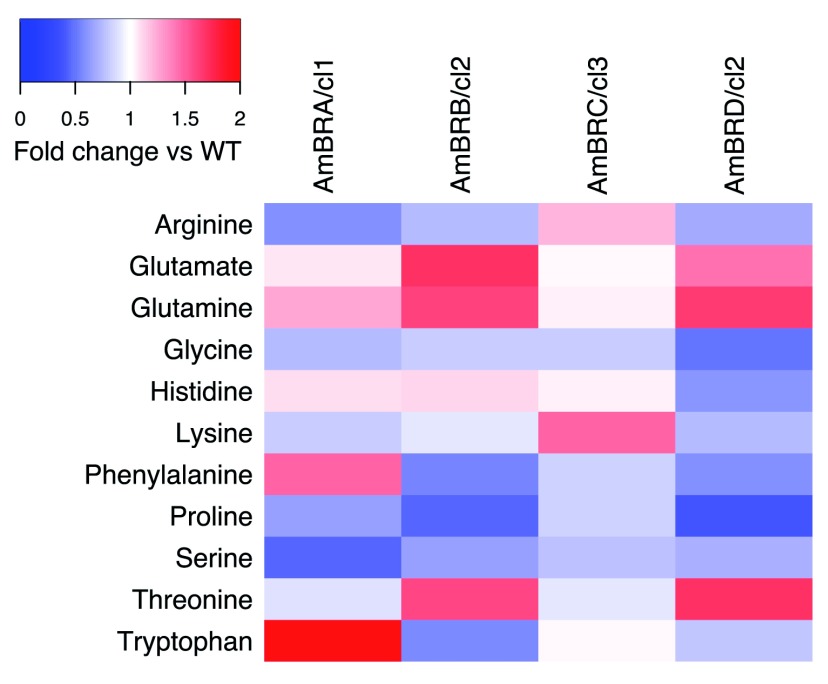
Heatmap of fold changes in amino acid intensity. Proteogenic amino acids with a Benjamini-Hochberg-corrected p-value (q-value) < 0.05 for ANOVA, and at least one Tukey’s Honest Significant Difference test comparing to wild-type with p-value < 0.05 are included (note that L- and D- isomers cannot be distinguished by this LC-MS method). Fold changes are in comparison with wild-type *L. mexicana*, with red representing increased abundance, and blue representing a decrease.

Of 319 metabolites putatively identified in this dataset, the largest annotated group was “amino acid metabolism” (101 metabolites). Of these, 66 showed significant differences from the parental line in at least one AmBR line, but overall the pattern was highly variable, including with respect to the 11 proteogenic amino acids identified ( Figure 4, note that while stereochemistry cannot be determined using this method, these are presumed L-amino acids as found in proteins). A previous study indicated that proline was increased in response to selection for resistance to multiple antileishmanial compounds in *L. donovani*, including AmB ^27^. Proline has previously been described as having ROS-scavenging activity ^28^. Here, however, there was a generalized trend towards deceased proline abundance (significant in AmBRB/cl2 and AmBRD/cl2), with no increases.

One of the most important molecules for defense against oxidative stress is dihydro-trypanothione, the reduced form of trypanothione that is oxidized to trypanothione disulfide in several ROS detoxification reactions. Two peaks were initially identified as trypanothione, one with a mass-to-charge ratio ( *m/z*) of 361.65 and retention time of 12.41 min, the other with an *m/z* of 723.30 and a retention time of 19.34 min. A peak for trypanothione was not found in the standards mix (despite this molecule being included), preventing differentiation on the basis of retention time. However, the 12.41 min peak was of higher quality, and moreover whilst the major *m/z* detected at 12.41 min was 361.65 (likely resulting from double ionization of trypanothione, mass 723.86), an additional *m/z* of 723.30 (likely resulting from single ionization) was also detected at this same retention time, supporting the 12.41 min peak as the true trypanothione peak. For both peaks, however, a strong increase in abundance was noted in AmBRA/cl1, AmBRC/cl3 and AmBRD/cl2. No change was observed in trypanothione disulfide, the more abundant form of trypanothione, suggesting that the reduced/oxidized trypanothione ratio was increased in these lines even if the overall trypanothione pool itself may not have greatly increased. However, spontaneous oxidation during metabolite extraction is feasible, and dedicated methods aimed at reducing redox changes during sample preparation would be required to probe redox status more deeply ^29^.

**Figure 5.**
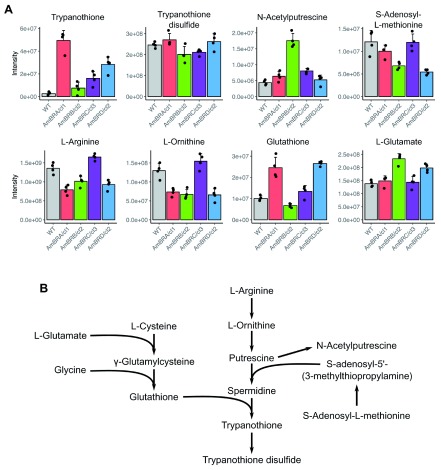
Changes in trypanothione biosynthesis. **A**) Metabolite intensity plots for metabolites involved in trypanothione biosynthesis. Mean intensities across four biological replicates are shown, with error bars representing standard deviation. Black points represent intensities of individual replicates. **B**) The trypanothione biosynthesis pathway.

Trypanothione arises from the ligation of the polyamine spermidine and two molecules of glutathione ( Figure 5). Polyamines themselves are poorly detected by the LC-MS system used. However, N-acetylputrescine, a derivative of the spermidine synthesis intermediate putrescine, did not show increases corresponding with those of trypanothione ( Figure 5). Likewise, S-adenosylmethionine, which is converted into S-adenosyl-5’-(3-methylthiopropylamine), the aminopropyl donor used to create spermidine from putrescine, showed decreased abundance in AmBRB/cl2 and AmBRD/cl2 ( Figure 5). Furthermore, arginine and ornithine, the upstream precursors of polyamine synthesis, actually showed decreased abundance in AmBRA/cl1, AmBRB/cl2 and AmBRD/cl2 ( Figure 5). Glutathione, the ROS-reactive thiol precursor of trypanothione, showed a similar, albeit more modest, pattern of increases in its reduced form to trypanothione itself ( Figure 5). Glutathione is a tripeptide of L-cysteine, L-glutamate and glycine. Of these, L-glutamate showed modest increases in AmBRB/cl2 and AmBRD/cl2, but overall, the pattern of increases did not match that of reduced glutathione. Unlike trypanothione, the disulfide (oxidized) form of glutathione was not detected. Therefore, whether the increase in reduced glutathione results from a more reduced state of the glutathione pool or an overall increase in its abundance cannot be determined.

## Discussion

Previously, to characterize the molecular basis of AmB resistance in *Leishmania* parasites, we selected resistance in four independent *L. mexicana* lines. In these lines, we determined a role for mutations in the genes sterol C5-desaturase ( *LmxM.23.1300*) and sterol C24-methyltransferase ( *LmxM.36.2380/LmxM.36.2390*), correlating with altered sterol profile ^15^. However, as changes in sterol profile did not fully explain differences in phenotypic traits, including virulence, we investigated broader metabolic alterations in these lines. Whilst numerous changes were observed, many of these were inconsistent between lines. This emphasizes the importance of testing multiple lines, since many of these changes may spontaneously arise during long term culture *in vitro*. Furthermore, the lack of an increase in proline metabolism differs from previous observations ^27^, although as these were made in *L. donovani* this may reflect species-specific differences in drug response. Overall, 11 metabolites showed differences from the parental wild-type conserved across all AmBR lines. However, these may reflect adaptation to long term culture, as the parental wild-type was isolated from an infected mouse shortly before selection began. To account for this, experiments can be performed comparing resistant lines to parasites cultured in parallel in the absence of drug. In this case, however, spontaneous changes arising in the wild-type during culture may further complicate analysis. Therefore, in the ideal scenario, both types of wild-type control (parental and long-term culture) should be used.

Overall, whilst numerous changes were observed, these were often modest in magnitude. One pathway that has previously been examined with respect to drug resistance in *Leishmania* is the pentose phosphate pathway ^20^. Theoretically, increased flux in this pathway could increase the NADPH pool, leading to a more robust defense against oxidative stress. Baseline abundance of PPP metabolites in the absence of drug does not appear to be an adaptation to AmB, although differences may emerge in the presence of drug-induced stress, such as a greater capacity to increase flux in response to AmB. In contrast large increases in both dihydro-trypanothione and reduced glutathione abundance were observed. Increases in the reduced thiol pool could result from either decreased consumption (due to lower baseline ROS generation) or increased reduction rate. For both molecules, reduction is an NADPH-dependent, cytosolic process. Hence the fact that NADPH is unchanged could suggest that production of NADPH is increased through a PPP-independent mechanism. A cytosolic malic enzyme has been described in *L. mexicana* ^30^, and would represent an alternative means of NADPH generation, although neither its substrate malate, nor its product pyruvate, showed altered abundance. Hence the mechanism leading to increased reduction of the thiol pool is unclear. Ovothiol, another redox-active thiol found in *Leishmania*, was detected but was not observed to increase in AmB-resistant lines.

Likewise, there is little evidence of broad changes in central carbon metabolism, and whilst there do appear to be general increases in lactate and succinate abundance, this could also be an adaptation to the high glucose content of the HOMEM culture medium used in this study. Nevertheless, given the documented importance of mitochondrial metabolism and ROS production in drug sensitivity ^23^, the balance of glycolytic and respiratory metabolism in drug-resistant *Leishmania* merits further investigation.

Another area of carbon metabolism showing substantial changes is in lipid composition. pHILIC LC-MS metabolomics is limited in its ability to resolve and identify lipid compounds. Nevertheless, these data reveal a number of changes specific to AmBRB/cl2, which carries a homozygous deletion in the phospholipid flippase *LmxM.13.1530*, responsible for AmB-miltefosine cross-resistance. Mutations in the *L. infantum* ortholog of this gene have previously been associated with AmB resistance ^13^, but the mechanism is unclear, although notably increased membrane fluidity is known to affect AmB activity ^31^. Given the potential for mutations in this gene to cause cross resistance to two major antileishmanial compounds, it will be useful to characterize this line further using a lipid-focused metabolomics method as previously described ^13^. High-throughput RNAi screening in *T. brucei* identified multiple flippase-encoding genes required for AmB sensitivity ^18^, strengthening the link between loss of flippase activity and AmB resistance.

An important observation from our previous study was that despite almost total loss of the dominant sterol in wild-type *L. mexicana*, ergosta-5,7,24(28)-trienol, and replacement with sterols lacking C24-methylation, the line AmBRC/cl3 retained wild-type infectivity both in primary macrophages and *in vivo* ^15^. In contrast, AmBRB/cl2 and AmBRD/cl2, lines exhibiting highly similar loss of sterol C24-methylation due to disrupted sterol C24-methyltransferase expression, showed lack of infectivity in macrophages, suggesting that loss of virulence arises through a mechanism unrelated to sterol changes. Mannogen, a carbohydrate storage polymer comprised of D-mannose (an epimer of D-glucose), is essential for virulence ^32^. Whilst larger polymers of mannose exceed the upper detected mass threshold of the LC-MS method used here, we observed consistent decreases in several oligo-hexoses (3-6 hexose units), as well as GDP-mannose, the mannose donor molecule during mannogen biosynthesis. Previously, we observed similar changes in a transketolase deletion mutant of *L. mexicana*, which were confirmed to have similar decreases in large molecular weight carbohydrate polymers ^33^. As this line was avirulent *in vivo*, this suggests that similar mannogen-related changes in AmBR lines could be responsible for lack of virulence. As mannogen is not required for growth *in vitro*, it is likely that these changes arose spontaneously during long term selection, unrelated to sterol changes. Whilst notably there are fewer changes overall in the virulent line, AmBRC/cl3, than in the other AmBR lines, nonetheless this suggests that loss of oligomannan content may be an important consideration in assessing virulence of parasite lines derived through similar methods.

Overall, therefore, these data suggest a picture where high variability arises largely in a stochastic fashion during experimental selection. The basis of this variability is not clear, although aneuploidy in these lines was correlated with widespread differences in steady-state gene expression ^15^, as similarly reported elsewhere ^34, 35^. This may have complex effects on the metabolome. Many of these changes are modest, and therefore unlikely to have strong effects on drug sensitivity. By contrast, increased trypanothione abundance is likely to have a stronger role in these lines, either in direct defense against drug-induced ROS or in supporting tolerance of resistance-related changes in sterol composition. On the other hand, spontaneous changes may impact virulence independent of the resistance mechanism itself, making assessment of the effects of resistance selection on virulence harder to interpret. As this is key to understanding the likelihood of such mutations emerging in the clinical context, it will be important to monitor for changes in pathways such as mannogen synthesis when determining the basis of virulence changes in drug resistant lines. As CRISPR-Cas9-based methods increase the feasibility of precise genome editing in *Leishmania spp.* ^36, 37^, it may be useful to replicate resistant-related mutations *de novo* on the parental background. A better understanding of the relationship between sterol profile and fitness is crucial to estimating the risk of emergent resistance associated with AmB-based treatment programs in the elimination of leishmaniasis as a public health threat.

## Methods

### Sample extraction

Derivation of AmB-resistant lines from parental *L. mexicana* M379 was described previously ^15^ (MNYC/BZ/62/M379 was originally isolated from Sumichrast’s vesper rat in 1962, Belize, and was a gift from Professor Graham Coombs). *L. mexicana* was cultured in HOMEM medium (Gibco cat. no 041-96499M) supplemented with 10% fetal bovine serum (Gibco cat. no. 10500-64) at 25 °C. Prior to sample extraction, parasite cell lines grown in the presence of AmB (Sigma cat. no. A2942) (400 nM for AmBRA/cl1 and AmBRB/cl2, 200 nM for AmBRC/cl3 and AmBRD/cl2; wild-type parasites were cultured in the absence of drug) were diluted to approximately 10 ^5^ parasites/ml and incubated for three days without AmB until parasites reached mid-log phase (5–10 × 10 ^6^ parasites/ml), at which point cells were counted and 10 ^8^ parasites were taken for metabolite extraction. Cultures were rapidly cooled to < 10 °C in a dry ice-ethanol bath with rapid agitation to avoid freezing, and samples were kept at 4 °C for the remaining stages of extraction, based on the method of t’Kindt and colleagues ^29^. Parasites were sedimented by centrifugation (1,250 × g, 10 min), resuspended in ice-cold PBS and sedimented again (1,900 × g, 10 min) before resuspension in extraction solvent (1:3:1 v/v/v chloroform:methanol:water). Extraction solvent was also added to empty tubes to act as blank samples. All samples were subjected to rapid agitation for 1 hr, before centrifugation at 17,000 × g, 10 min to remove cellular debris. Supernatants were retained and stored at -80 °C under argon prior to analysis. Four replicates were obtained from independently grown cultures for each cell line. A pooled sample was produced as a quality control containing equal amounts of each sample, topped up with further extraction solvent.

### Liquid chromatography-mass spectrometry

LC-MS analysis was performed at Glasgow Polyomics. Separation of metabolites was performed by hydrophilic interaction liquid chromatography (HILIC) using the Dionex UltiMate RSLC system (Thermo Fisher Scientific) with a ZIC-pHILIC column (Merck Sequant). Column temperature was maintained at 30 °C during separation, with elution of samples using a linear gradient between two mobile phase solvents, solvent A (20 mM ammonium carbonate in water) and solvent B (acetonitrile). An initial A:B solvent ratio of 20:80 was gradually altered to 80:20 over a 15 min period, after which the gradient was changed to 95:5 as rapidly as possible and held for 2 min. After returning the gradient to 20:80 as rapidly as possible, this was held for a further 7 min. The flow rate was 0.3 ml/min, with a 10 μl injection volume. Samples were maintained at 4 °C prior to injection. Sample order was randomized, with pooled sample run every four samples to allow for assessment of instrument reproducibility over time.

Mass spectrometry was performed using a Thermo Orbitrap Exactive (Thermo Fisher Scientific), operating in polarity switching mode. The following settings were used: the resolution was 50,000, the *m/z* ratio was 70–1,400, the automatic gain control target was 10 ^6^, the probe temperature was 150 °C and the capillary temperature was 275 °C. Flow rates were as follows (in arbitrary units): 40 for sheath gas, 5 for auxiliary gas, 1 for sweep gas. Samples were ionized by electrospray ionization (ESI). Positive mode ionization was performed with a source voltage of +4.5 kV, a capillary voltage of +50 V, a tube voltage of +70 V, and a skimmer voltage of +20 V. Negative mode ionization was performed with a source voltage of -3.5 kV, a capillary voltage of -50 V, a tube voltage of -70 V, and a skimmer voltage of -20 V. Mass calibration was performed with each polarity prior to every analysis batch using Thermo Calmix standards for *m/z* < 1,400 (mixture of Pierce LTQ Velos ESI Positive Ion Calibration Solution, cat. no. 88323, and Pierce Negative Ion Calibration Solution, cat. no. 88324), with additional inclusion of ubiquitous low-mass contaminants (C _2_H _6_NO _2_, m/z 76.0393, for positive ion ESI and C _3_H _5_O _3_, m/z 89.0244, for negative ESI). These low-mass contaminants were used to apply lock-mass correction to each analytical run, to enhance calibration stability.

### Data analysis

Initial metabolite identification from raw data, and relative quantification using peak area, were performed using the mzMatch (v2.0) package in R ^38^ and IDEOM software (v19) ^39^. Putative metabolite identification corresponds for the most part to Metabolite Standards Initiative (MSI) level 2 (mass only), whereas metabolites matching in retention time to an included standard correspond to level 1, as indicated in the underlying data ^22, 40^. Peaks having an area with root squared deviation across pooled samples > 50% were excluded, as were those with a retention time < 4 min (due to poor resolution) those identified as peptides (due to poor reproducibility), and those not matched to a metabolite. Peaks were subjected to initial filtering in which poor quality peaks were manually removed (performed in a blinded fashion in which metabolite names were unknown). Data were then log-transformed (log _10_(intensity + 1)) and a one-way ANOVA test was performed, followed by a Tukey’s Honest Significant Difference *post hoc* test to compare sample groups. A Benjamini-Hochberg multiple comparisons procedure was used to adjust p-values from the initial ANOVA. All statistical analysis was performed in R (v.3.4.4), and plots were generated using ggplot2 (v3.1.1) ^41^ and gplots (v3.0.1.1) ^42^ R packages.

## Data availability

### Underlying data

Raw data for the metabolomics experiment along with assay details, Accession number MTBLS1167: https://identifiers.org/metabolights:MTBLS1167

Full data for identified metabolites in wild-type and amphotericin B-resistant *Leishmania mexicana* parasites, figshare: https://doi.org/10.6084/m9.figshare.10059710.v1
