# Supplementary material for: Untargeted metabolomics to understand the basis of phenotypic differences in amphotericin B-resistant Leishmania parasites
Source: Wellcome Open Res. 2019 Nov 13;4:176. [Version 1] doi: 10.12688/wellcomeopenres.15452.1 (PMC7041363; doi:10.12688/wellcomeopenres.15452.1)
Supplement: Peer review history

# Peer review: Reviewer response for version 1

**DOI:** 10.21956/wellcomeopenres.16898.r37026

**Published:** 2020-02-20

**Authors:** Michael L. Ginger

**Affiliations:** 1 School of Applied Sciences, University of Huddersfield, Huddersfield, UK

Amphotericin B (AmB) is used in the treatment of the tropical disease leishmaniasis. Through its interaction with ergosta-type sterols, AmB disrupts membrane function in *Leishmania* parasites. In previous work the corresponding author’s team and other researchers have independently shown that mutation in any of several gene loci encoding different enzymes required for sterol ring or side-chain modification(s) leads to altered sterol compositions and links strongly to selection of AmB-resistant parasites. However, other genetic alterations have also been associated with decreased sensitivity to AmB and in laboratory-selected ‘sterol-altered’, AmB-resistant cell lines differences in parasite infectivity to macrophages or virulence within mouse models have been reported. Thus, in the current work Barrett and Pountain used metabolomics to study changes in cellular metabolism in four independently-selected AmB-resistant cell lines for which the same group had characterised phenotype, genotype and transcriptome relative to ‘wild type’ parent.

 Whilst the results from Pountain’s metabolomics analysis provide little evidence, at this point, for central metabolic changes (outside of sterol metabolism) consistently associated with AmB resistance or which adequately explain phenotype variations between mutants, this is an interesting data set for those working (or seeking to work) in the area(s) of trypanosomatid metabolism and drug resistance. For instance, the current health burden from leishmaniasis is such that new medicine development and insight into how human-infectious *Leishmania* species generally readily develop drug-resistant phenotypes are critical. Over many years Barrett’s team have pioneered the development of robust procedures for metabolite extraction and analysis from trypanosomatid parasites; their approaches thus, provide an excellent basis for others to standardise their metabolomics methods against. Similarly, it could be pertinent to determine how often changes in carbohydrate abundance are associated with decreased virulence. The open access presentation of datasets, such as the current one, should only therefore help in cost-effective new medicine development for leishmaniasis.

I confirm that I have read this submission and believe that I have an appropriate level of expertise to confirm that it is of an acceptable scientific standard.

# Peer review: Reviewer response for version 1

**DOI:** 10.21956/wellcomeopenres.16898.r37024

**Published:** 2019-11-29

**Authors:** Marc Ouellette

**Affiliations:** 1 Department of Microbiology, Infectious Disease and Immunology, Laval University, Québec City, Canada

Poutain and Barrett have carried out a metabolomic study of amphotericin B resistance in Leishmania. They studied 4 mutants that were previously sequenced. Three genes were associated with resistance but the resistance levels could not be fully explained by these mutations hence this metabolomic study in order to better understand resistance.

 This lab is a world leader in metabolomics studies. In this study, however, this approach was only moderately useful. Yet it is important to report since others who may have been interested by this approach may think about something else for studying AMB resistance. Indeed, Wellcome Open Research will publish those types of studies.

 This manuscript provides evidence that metabolite abundance is very cell line dependent and not related to the mode of action of the drug. Is that the norm or something special related to either the parasite or the drug used?

 The authors indicate that metabolites related to lipid metabolism showed altered abundance in particular in the B mutant with the miltefosine transporter deleted. This is of interest and additional studies (e.g. by manipulating the MT gene in a cleaner background) may help in studying lipid metabolism in the context of AmB resistance.

I confirm that I have read this submission and believe that I have an appropriate level of expertise to confirm that it is of an acceptable scientific standard.
